# Molecular spectroscopies with semiconductor metasurfaces: towards dual optical/chemical SERS

**DOI:** 10.1039/d4tc05420b

**Published:** 2025-05-22

**Authors:** Alexander Berestennikov, Haiyang Hu, Andreas Tittl

**Affiliations:** a Chair in Hybrid Nanosystems, Nanoinstitute Munich, Faculty of Physics, Ludwig-Maximilians-Universität München 80539 München Germany andreas.tittl@physik.uni-muenchen.de

## Abstract

Surface-enhanced Raman spectroscopy (SERS) has emerged as a powerful technique for the ultra-sensitive detection of molecules and has been widely applied in many fields, ranging from biomedical diagnostics and environmental monitoring to trace-level detection of chemical and biological analytes. While traditional metallic SERS substrates rely predominantly on electromagnetic field enhancement, emerging semiconductor SERS materials have attracted growing interest because they offer the additional advantage of simultaneous chemical and electromagnetic enhancements. Here, we review some of the recent advancements in the design and optimization of semiconductor SERS substrates, with a focus on their dual enhancement mechanisms. We also discuss the transition from nanoparticle-based platforms to more advanced nanoresonator-based SERS metasurfaces, highlighting their superior sensing performance.

## Abstract 2

Semiconductor metasurfaces enable dual SERS enhancement by combining strong optical resonances with charge-transfer interactions, offering a powerful platform for highly sensitive molecular detection.

## Graphical abstract

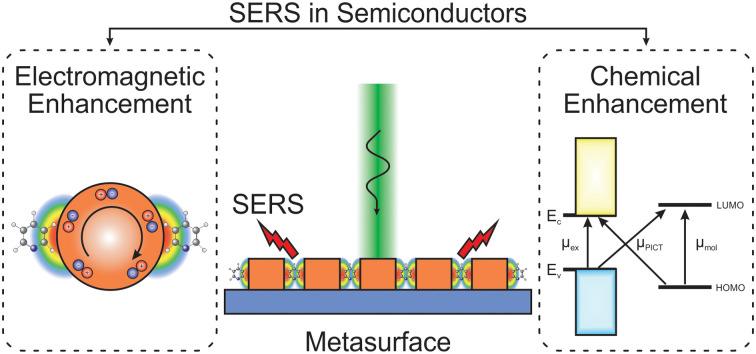

## 1. Brief overview of surface enhanced Raman scattering

SERS has emerged as a state-of-the-art analytical methodology and provides a sensitive, selective and non-destructive tool for bio- and chemical sensing.^1–8^ Its potential spans diverse fields including medical diagnostics, environmental monitoring, and food safety, prompting a surge of interest in both the development of novel SERS-active materials and the optimization of its real-world applications over the past decade.^9–14^ At its core, Raman spectroscopy is based on the phenomenon of inelastic photon scattering, a concept initially proposed by Smekal^15^ in 1923 and later validated experimentally by Raman and Krishnan in 1928.^16^ Within this scattering process, the vibrational or rotational modes of molecules undergo excitation or relaxation, resulting in a wavelength shift of scattered photons. This allows for the collection of unique Raman spectra for each chemical, correlated with their distinct vibrational energy levels. However, conventional Raman spectroscopy faces limitations due to its inherently low scattering cross-section compared to other spectroscopic methods such as ultraviolet (UV), infrared (IR), and fluorescence spectroscopy, thereby constraining its applicability in resolving molecular systems with low concentrations or extremely small thicknesses.

**Fig. 1:**
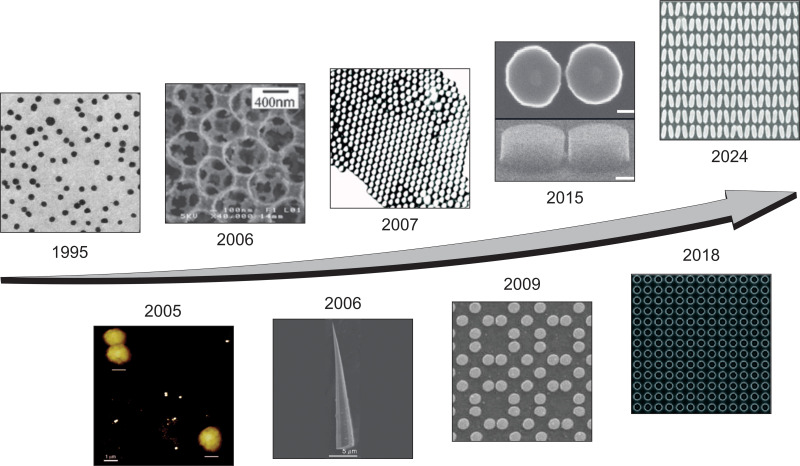
Advances in fabrication of metal and semiconductor SERS substrates. The progression illustrates the transition from simple colloidal particles to complex metasurfaces with precise nanoscale patterns, enabling enhanced Raman signal detection and broad application potential. (1995) Adapted with permission from ref. 17. Copyright 1995, AAAS. (2005) Adapted with permission from ref. 19. Copyright 2005, American Chemical Society. (2006) Adapted with permission from ref. 21. Copyright 2006, the Royal Society of Chemistry. (2009) Adapted with permission from ref. 22. Copyright 2009, American Chemical Society. (2006) Adapted with permission from ref. 30. Copyright 2006, the Royal Society of Chemistry. (2007) Adapted with permission from ref. 31. Copyright 2007, AIP Publishing. (2015) Adapted with permission from ref. 32. Copyright 2015, Springer Nature. (2018) Adapted with permission from ref. 33. Copyright 2018, American Chemical Society. (2024) Adapted with permission from ref. 34. Copyright 2024, John Wiley and Sons.

The evolution of SERS substrates has been transformative (Fig. 1). Following the general development path of nanophotonics, metals such as Ag, Au, and Cu became the first materials for SERS platforms.^17–20^ The ability of electromagnetic radiation to interact with metal particles whose size is much smaller than the wavelength of incident radiation induces coherent oscillations of the electrons in the material, called plasmons. Metal nanoparticles (NPs), while effective, initially suffered from limitations in reproducibility and stability, as their enhancement factors were highly dependent on uncontrolled aggregation and morphology. Building on these initial discoveries, researchers developed ordered metallic nanostructures, photonic crystals, and metasurface platforms to overcome these challenges.^21,22^ Metallic nanostructures can exhibit diverse plasmonic modes such as localized surface plasmon resonances (LSPRs) and propagating surface plasmon polaritons (SPPs), which can lead to significant enhancement of the local electromagnetic fields, resulting in unrivaled improvements in SERS performance. By leveraging the principles of nanophotonics, metasurface-based SERS substrates provide tunability, enabling tailored enhancement profiles for specific analytes and applications.^18,23–26^ Moreover, the integration of functional materials and novel geometries has expanded the versatility of metasurface substrates, opening new ways for multifunctional and label-free sensing applications.^27–29^

In parallel with advancements in metallic SERS platforms, significant progress has been made in the development of semiconductor-based SERS substrates.^30–35^ Since the observation of the SERS effect on GaP semiconductor NPs in 1988, there has been increasing attention in extending the range of semiconductors available for SERS.^36,37^ The use of various semiconductor materials for SERS has been well reviewed in the literature.^37,38^ It has been shown that chemical enhancement (CE) is an important contribution to SERS, given that it is difficult to achieve strong electromagnetic enhancement (EE) comparable to the values commonly found in plasmonic systems. However, the development of nanophotonics has allowed the creation of semiconductor nanostructures having various optical resonances, such as Mie resonances, which has enabled high EE values to be achieved.^39–41^ These platforms allow the flexible use of both mechanisms for enhancing Raman signals, relying not only on its original CE but also on the additional light confinement based on optical resonances.^42–44^ Additionally, semiconductor substrates provide several advantages, like convenient surface functionalization as well as superior chemical and thermal stabilities compared to metallic systems, making them highly suitable for applications in complex environments.^37,45–48^ It can open up opportunities for highly sensitive, reproducible, and multifunctional SERS substrates, with applications ranging from molecular diagnostics to environmental sensing and catalysis.

## 2. SERS mechanisms

Currently, two complementary theories explaining the mechanism of SERS enhancement are widely accepted: EE and CE.^26,37,49–51^

### 2.1. Mechanism 1: electromagnetic enhancement

**Fig. 2:**
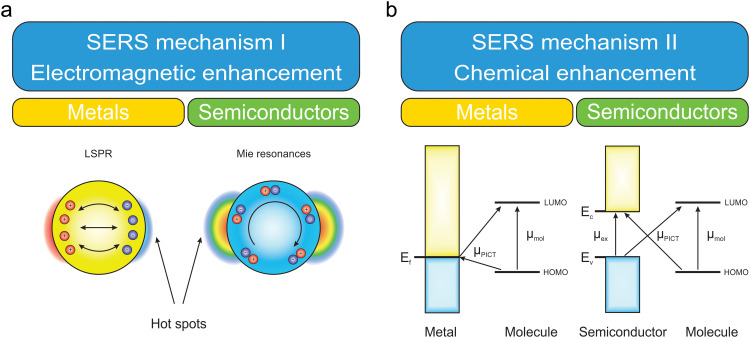
SERS enhancement mechanisms: (a) electromagnetic enhancement illustrated by LSPRs in metals and Mie resonances in semiconductors. (b) Chemical enhancement illustrating CT interactions between the metal or semiconductor energy bands and the molecular energy levels (HOMO and LUMO).

EE stands out as a major contributor to SERS.^52,53^ This phenomenon is based on the amplification of the electromagnetic field *via* the excitation of different optical resonances within the SERS-active substrate. In metals, the optical mechanism is predominantly driven by the excitation of LSPRs, which occur when incident light interacts with the free electrons of metallic nanostructures, causing a collective oscillation of these electrons at specific resonant frequencies.^54,55^ LSPRs amplify the local electromagnetic fields near the metal surface, creating regions known as “hot spots”, which are typically located at sharp edges, narrow gaps, or irregularities on the metal nanostructure (Fig. 2a).^56,57^ These fields enhance the interaction between the incident light and analyte molecules adsorbed on the surface. The role of LSPRs is to amplify both the incident electromagnetic field during excitation and the scattered field during emission, leading to a total enhancement proportional to ∣*E*∣^4^.^26,58,59^ Additionally, the resonance frequency of LSPRs can be tuned by altering the size, shape, and arrangement of the nanostructures, allowing optimal enhancement for specific excitation wavelengths.^19,28,60,61^ The efficiency of the optical mechanism in metallic SERS substrates is also affected by factors like the dielectric environment, interparticle coupling in aggregates, and the intrinsic losses due to electron damping.

The electromagnetic SERS enhancement in semiconductors has the same mechanism as in metals, but instead of using LSPRs, it leverages alternative light–matter interaction mechanisms such as Mie resonances and other photonic resonances.^39,42–44^ Semiconductor materials, such as Si, Ge, TiO_2_, ZnO and others, have high refractive indices and low optical losses, enabling the confinement and amplification of electromagnetic fields through scattering and interference effects. For example, Mie resonances occur when the dimensions of semiconductor nanostructures are comparable to the wavelength of incident light, leading to constructive interference and localized field enhancement near the particle surface. These effects can amplify the Raman scattering signal of molecules.^49^ One recent development in all-dielectric nanophotonics is bound states in the continuum (BICs), which can arise from symmetry-protection, destructive interference, or parameter tuning, creating non-radiative electromagnetic modes with exceptionally high-quality (*Q*) factors and intense field localization.

Although the EE in semiconductors is typically weaker than that in metals, semiconductor SERS substrates, through the integration of advanced fabrication technologies, can provide a significant field enhancement while maintaining excellent chemical and thermal durability, making them promising alternatives for sensing, imaging, and environmental monitoring.^51^

### 2.2. Mechanism 2: chemical enhancement

Despite offering insights into the origin of Raman signal enhancement, the electromagnetic theory struggles in explaining the magnitude of observed amplification.^37,51^ The CE theory offers a complementary, but fundamentally distinct role from the EE mechanism, focusing on the creation of charge-transfer (CT) states between the adsorbed molecule and the SERS platform.

The substrate-related CE originates primarily from three contributions: molecular resonances, CT resonances between the molecular levels of the analyte and the Fermi level (or band edges in the case of semiconductors) of the substrate, and nonresonant modification of molecular polarizability.^37,51^ The first contribution can be achieved, when the excitation laser frequency is chosen at a molecular resonance. The latter mechanism occurs due to formation of a substrate–molecular complex, consequently, distortion of the molecular electron cloud. Adsorption creates a static change in the molecular polarizability, altering its electronic structure and enhancing its response to external electromagnetic fields. This process affects the ability of the molecule to participate in CT processes. As described in Lombardi's theoretical framework,^37^ the CT transitions occur, when the energy levels of the molecules and the metal (or semiconductor) substrate are coupled, allowing electrons to move between the molecule and the substrate. The strongest Raman enhancement in this CT complex can be achieved if the excitation frequency resonates with the photon-induced charge transfer (PICT) transition in this system (Fig. 2b). For metal substrates, depending on the relative position of the Fermi level, and the HOMO/LUMO levels of the molecule, this CT can occur in the direction of molecule-to-metal or metal-to-molecule. In the case of semiconductors, the CT scheme is similar to that in metals, but the CT occurring between the valence/conduction bands of semiconductors, and LUMO/HOMO levels in the molecule. The scheme for semiconductors also includes the possibility of the exciton resonance.

Thus, the chemical mechanism is essential for explaining the selective enhancement of specific vibrational modes and the sensitivity of SERS to molecular bonding environments. Furthermore, it extends SERS to analytes with poor polarizability or limited affinity for non-functionalized metal surfaces.

## 3. Chemically active semiconductor materials for SERS

The use of semiconductor materials in SERS nanostructures enables the synergistic combination of EE and CE mechanisms in advanced SERS applications.^51^ Selecting a suitable semiconductor is a fundamental step, since its intrinsic properties – such as the refractive index, bandgap properties, and surface chemistry – directly influence the efficiency of both resonance-driven EE and CE interactions with analyte molecules.

### 3.1. SERS active semiconductor materials

In this section, we review semiconductor materials and their properties, which can be beneficial for the CE and EE mechanisms. When designing nanostructures based on these materials, the refractive index (*n*) and extinction coefficient (*k*) must be considered. A high refractive index is preferable to create optical resonances, while too high *k* results in their significant damping, which reduces the overall efficiency.^62,63^Table 1 summarizes the *n* and *k* values for various semiconductor materials commonly used in SERS at different wavelengths (532 nm, 633 nm, 785 nm, and 1064 nm), corresponding to the most widely used Raman excitation sources.^51^ All data were extracted from literature sources, with references provided in the last column.

**Table 1:** Summary of refractive indices (*n*) and extinction coefficients (*k*) for semiconductors and dielectric materials for SERS. All data were extracted from literature sources, with references provided in the last column

Material	532 nm		633 nm		785 nm		1064 nm		Ref.
	*n*	*k*	*n*	*k*	*n*	*k*	*n*	*k*	
TiO_2_	2.45	<0.01	2.39	<0.01	2.34	<0.01	2.31	<0.01	64
ZnO	1.8	0.06	1.77	0.04	1.76	0.03	1.74	0.02	65
V_2_O_5_	2.59	0.12	2.47	0.05	2.40	0.03	2.33	0.02	66
WO_3_	2.00	0	1.97	0	1.96	0	1.94	0	67
Cu_2_O	9.14	<0.01	3.48	<0.01	2.79	<0.01	2.55	<0.01	68
MoO_3_	1.86	0.01	1.82	<0.01	1.79	<0.01	1.7	<0.01	69
Nb_2_O_5_	2.41	0	2.36	0	2.33	0	2.29	0	70
Fe_2_O_3_	2.97	0.43	2.76	0.31	2.46	0.29	2.13	0.30	71
ITO	1.87	0.01	1.76	0.01	1.57	0.03	1.07	0.1	72
Ge	4.95	2.35	5.46	0.74	4.72	0.29	4.38	0.15	73
Si	4.14	0.03	3.87	0.02	3.69	0.01	3.55	<0.01	74
Si_3_N_4_	2.03	0	2.02	0	2.01	0	2.00	0	75
GaN	2.34	0.07	2.29	0.06	2.25	0.05	2.22	0.04	76
GaP	3.51	0.05	3.33	0.01	3.19	0	3.10	0	77
PbS	3.55	1.18	3.8	1.08	3.66	0.54	3.44	0.15	78
PbSe	5.02	2.75	4.87	1.93	5.13	1.79	5.17	0.79	79
SnS_2_	2.97	0.03	2.83	0	2.75	0	2.96	0	80
SnSe_2_	3.69	0.56	3.50	0.28	3.30	0.13	3.13	0.04	80
ZnS	2.42	0.04	2.37	0.03	2.33	0.03	2.29	0.02	81
ZnSe	2.67	0.07	2.54	0.06	2.47	0.04	2.46	<0.01	82 and 83
CdS	2.55	0.01	2.38	0	2.31	0	2.26	0	84
CdSe	2.54	0.44	2.59	0.32	2.52	0.10	2.38	0.07	85
Graphene	2.72	1.34	2.74	1.4	2.80	1.51	2.90	1.80	86
MoS_2_	5.22	0.98	5.50	1.28	4.69	0.02	4.24	0	87
WS_2_	5.06	0.76	5.21	1.17	4.16	0	3.88	0	87
ReS_2_	4.48	1.36	4.63	1.11	4.59	0.46	4.19	0.01	87
MoSe_2_	5.06	1.91	5.02	1.11	4.87	0.79	4.48	<0.01	87
WSe_2_	4.51	1.28	4.90	0.61	5.20	0.38	4.19	0	87
hBN	2.21	0.01	2.18	0.01	2.15	0.01	2.13	<0.01	88

Among SERS-active semiconductors, metal oxides represent a diverse class with unique optical, electronic, and chemical properties that make them highly attractive for Raman signal enhancement.^89,90^ TiO_2_ is one of the most studied and promising materials for SERS, showing many advantages such as high chemical stability, versatile surface functionalization, photocatalytic recyclability, and biocompatibility.^34,91–99^ TiO_2_ has a high refractive index (*n* ≈ 2.5) and low intrinsic losses in the visible range, which have attracted great attention from the SERS community.^34^ At the same time, TiO_2_ shows the possibility to form electronic states that interact with adsorbed molecules (especially due to oxygen vacancies or defects), thereby enabling CT processes that lead to effective CE.^96,100,101^ Most of the aspects discussed for TiO_2_ are also applicable to another widely used SERS semiconductor, ZnO.^51,102,103^ It has a slightly lower refractive index (*n* ≈ 1.8–2.0 in the visible range), but due to its low absorption this material is suitable for creating resonant nanostructures. It was also shown that ZnO can exhibit strong dual SERS enhancement with a maximum EF of ∼10^5^.^50^ Examples of CE in ZnO can be found in several works reported by different groups.^50,104–107^ There are many other metal oxide semiconductors that have not been as widely studied for SERS applications, such as Cu_2_O, V_2_O_5_, WO_3_, MoO_3_, Fe_2_O_3_, ITO, and Nb_2_O_5_.^108–116^ However, they show promising material properties beneficial for SERS enhancement. Notably, Nb_2_O_5_ has been identified as one of the most efficient semiconductor SERS materials, achieving an EF of ∼10^7^, which is close to that of traditional metal substrates.^115^

Classic covalent semiconductors like Si and Ge as well as compound semiconductors like Si_3_N_4_, GaP, GaN, ZnS, ZnSe, CdS, CdSe, PbSe, PbS, SnSe_2_, and SnS_2_ have also demonstrated SERS activity.^36,38,51,117–129^ These materials typically have high refractive indices in the visible and near-IR regions (*n* ≈ 3–4). Si, Ge, GaP, and GaN SERS substrates usually rely mostly on the EE mechanism, exhibiting weaker CE compared to metal oxides (unless they are further functionalized).^130^ As a result, most of them are commonly paired with plasmonic materials to boost their SERS performance.^117,131,132^ The main advantage of this class of materials is their good compatibility with nanofabrication methods such as photo- and electron lithography. By contrast, ZnSe, CdSe, PbSe, PbS and others show a significant CE contribution to SERS enhancement.^120,123,129^ This happens due to the fact that exciton and/or CT interband transitions occur between the material and the adsorbed molecule.^37^ However, in most cases, only quantum dots were used. This limited the enhancement mechanism only to CE due to the small particle sizes, which is insufficient for optical resonances to occur. Thus, further studies investigating the dual mechanism in this class of materials are needed.

Two-dimensional (2D) materials have emerged as a promising new class of semiconductors for SERS.^133–151^ van der Waals (vdW) materials such as graphene, transition metal dichalcogenides (TMDCs) (*e.g.*, MoS_2_, WS_2_, MoSe_2_, WSe_2_, and ReS_2_), and hBN offer tunable bandgaps, high surface-to-volume ratios, and strong CT interactions.^135,136,138,146,152–155^ The main contribution to the SERS enhancement in 2D materials is made by CE mechanisms.^146,155,156^ For example, graphene's CE arises from CT resonance, which leads to a maximum EF of ∼10^2^.^143,155^ This is akin to non-plasmonic SERS theory as per Lombardi's model, except here the graphene's continuous band (zero-gap) means it can interact over a broad range.^37,51^ By tuning its Fermi level (*via* gating or doping), one can modulate the intensity of the Raman signal of adsorbed molecules.^157,158^ Experiments have shown that applying a gate voltage to graphene can either enhance or suppress Raman peaks of adsorbed molecules depending on the alignment of levels, confirming the CT mechanism. Another vdW material, hBN, is a wide-bandgap insulator with a band gap of about 5.97 eV and *n* ≈ 2.1. It also relies on the CE mechanism, but unlike graphene, the enhancement occurs due to a strong dipole–dipole interaction between the monolayer and the adsorbed molecule, since hBN has a polar structure.^134^ In turn, TMDCs are the rising stars for semiconductor nanophotonics.^159–162^ They provide CE similar to traditional semiconductors, and combine the enhancement mechanisms of graphene and hBN:CT and dipole–dipole interaction.^134–138,140,145,156^ Recently, it was shown that defect states in TMDCs can play a significant role in CE, and due to this, high EF values up to ∼10^5^–10^6^ can be achieved for these materials.^147^ Electromagnetic field enhancement from an isolated 2D sheets is usually weak since no hotspots are formed on a pure monolayer unless it is nanostructured. To improve their efficiency and versatility, various 2D material-based SERS substrates with metal NPs have been developed.^163–170^ To obtain fully semiconductor and efficient 2D SERS substrates, a method for creating multilayer heterostructures from 2D material sheets has been proposed.^139,153,171^ Such structures have already been used to fabricate nanoantennas and metasurfaces with high-*Q* optical resonances.^172–178^ However, they have not been investigated in SERS applications so far and may be promising candidates for next-generation SERS substrates.

### 3.2. Tuning chemical enhancement

Semiconductor energy band engineering provides a powerful approach to improve the CE mechanism of SERS. Proper alignment of the CB or the VB with the analyte's LUMO and HOMO levels provides energetically favorable CT pathways, resulting in enhanced Raman scattering. Two common methods for achieving this are the introduction of oxygen vacancies and material doping.^179–181^

**Fig. 3:**
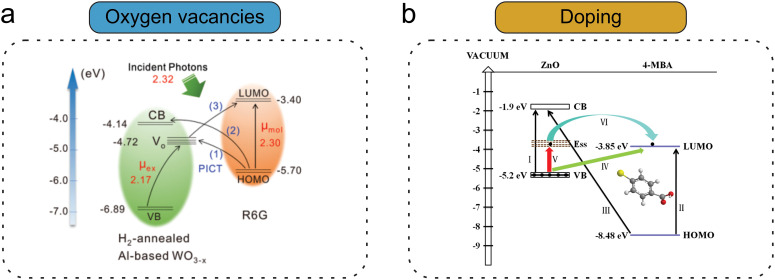
Strategies for enhancing CT mechanisms in semiconductor-based SERS substrates: (a) creation of oxygen vacancies, where vacancy states facilitate PICT between the CB and the molecular HOMO/LUMO levels. (b) Material doping illustrated by doping of ZnO NPs with Ga atoms, which adjusts the energy band alignment to optimize CT and enhance SERS activity. (a) (2019) Adapted with permission from ref. 112. Copyright 2019, John Wiley and Sons. (b) (2019) Adapted with permission from ref. 183. Copyright 2019, Frontiers Media SA.

Fan *et al.*^112^ achieved improved SERS performance using oxygen vacancy-rich WO_3−*x*_ as a substrate due to the enhanced PICT resonance originated from the additional oxygen vacancy levels in the bandgap (Fig. 3a), stronger exciton resonance, and higher electronic density of states near the Fermi level. This resulted in efficient CT pathways as well as enhanced vibronic coupling between analytes and SERS substrates. A similar mechanism was also applied for TiO_2_.^96^ Enhanced SERS performance was also observed through oxygen incorporation into the SERS-active MoS_*x*_O_*y*_ semiconductor substrates.^182^

Doping of semiconductors provides a convenient way to adjust the band gaps and add new levels in the band gap, which also facilitate the CT process, and can improve the SERS activity (Fig. 3b). Specifically, doping-generated defects help in increasing the charge carrier density by transferring electrons or holes.^184^ In the past few years, extensive research has been carried out focusing on improving the SERS performance of semiconductors by doping with various metal or non-metal materials.^183,185–190^

## 4. Optical resonances for SERS in semiconductors

Following material selection, the implementation of optical resonators tailored to the desired spectral range further amplifies local fields. By engineering the metasurface geometry at the nanoscale, these resonators can be precisely structured to optimize light confinement, enhance molecular interactions, and facilitate strong signal amplification.

### 4.1. Mie resonances

**Fig. 4:**
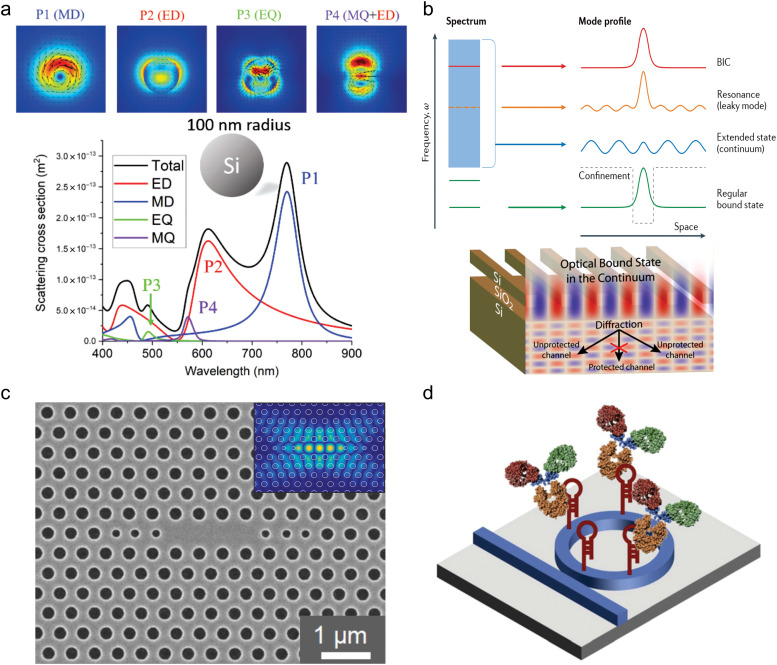
Overview of advanced semiconductor-based metasurfaces for SERS applications: (a) scattering cross-sections of silicon nanospheres demonstrating multipole resonances (ED: electric dipole, MD: magnetic dipole, EQ: electric quadrupole, and MQ: magnetic quadrupole) and their contributions to SERS enhancement. (b) Bound states in the continuum (BIC) in optical systems, showing protected and unprotected channels for enhanced light–matter interactions. (c) SEM image of the gallium nitride L3 photonic crystal cavity, illustrating their potential for optical confinement and field enhancement. (d) Schematic representation of a label-free aptamer sensor based on silicon microring resonators, highlighting its functional application in SERS-based molecular detection. (a) (2020) Adapted with permission from ref. 191 Copyright 2020, De Gruyter. (b) (2016) Adapted with permission from ref. 43. Copyright 2016, Springer Nature. (2017) Adapted with permission from ref. 196. Copyright 2017, American Chemical Society. (c) (2014) Adapted with permission from ref. 197. Copyright 2014, AIP Publishing. (d) (2013) Adapted with permission from ref. 198. Copyright 2013, Elsevier.

Mie resonances, arising from the interaction of light with semiconductor particles of subwavelength dimensions, play a significant role in enhancing SERS signals. They arise from constructive interference of scattered light within the particle and can be observed for high-refractive-index materials, such as Si, TiO_2_, or ZnO. These resonances allow the confinement and enhancement of electromagnetic fields near and inside the NP (Fig. 4a), providing a low-loss and high-*Q* alternative to traditional plasmonic structures, where damping effects lead to broader and less defined spectral features.^39,49,62,63,191–195^

For SERS applications, the careful engineering of the particle size, geometry, and arrangement is critical for optimizing the resonance conditions.^199–202^ Spherical NPs, nanodisks, and nanorods are commonly employed morphologies, with their dimensions tailored to achieve resonances at specific excitation wavelengths.^32,35,63,203^ Arrays of semiconductor particles can further enhance Mie resonance effects through collective interactions, creating uniform field distributions and improving reproducibility across the substrate.

Moreover, Mie resonances can synergize with semiconductor bandgap engineering to improve the CE contribution to SERS. By selecting materials with appropriate refractive indices and absorption properties, it is possible to design substrates that simultaneously support strong EE *via* Mie resonances and facilitate CT interactions with analyte molecules.^50^

### 4.2. BIC resonances

BIC resonances have emerged as a novel and highly effective mechanism for enhancing Raman signals in SERS.^42,204–209^ The mechanism of BIC relies on the creation of non-radiative electromagnetic modes that remain localized within a structure, even though they coexist with a continuum of radiative states. These non-leaky states result from specific symmetries or interference effects in the system, which prevent energy leakage into the far field. BICs are characterized by extremely high-*Q* factors, as they do not couple with radiative modes (Fig. 4b).^42,43,196,204,210,211^ This unique property allows the generation of highly confined electromagnetic fields with significantly enhanced intensity.^33,34^

Supported by semiconductor nanostructures BIC resonances allow the precise control of light–matter interactions through careful structural and material design.^44,212^ Unlike plasmonic substrates prone to dissipative losses, BIC systems achieve superior field localization and extended interaction times due to their negligible radiative decay. By offering low-loss, tunable, and highly reproducible enhancement mechanisms, BIC-based substrates represent a major advancement in SERS platform design. Their ability to detect low-concentration analytes with precision expands the potential of SERS in chemical sensing, biosensing, and environmental monitoring, providing a stable and versatile solution for advanced analytical applications.^213–216^

### 4.3. Other photonic resonances

In addition to structures that support Mie and BIC resonances, other photonic architectures have shown promising SERS activity. Photonic crystals, whispering-gallery mode (WGM), and waveguide resonators provide alternative ways for the field enhancement through the control of light confinement and propagation within semiconductor materials.

Photonic crystals, with their periodic lattices, create photonic bandgaps that prevent the propagation of light within certain frequency ranges. By introducing structural defects into these periodic systems, localized modes can form, enabling intense light confinement and enhanced electromagnetic fields near the defect region (Fig. 4c).^197,217,218^ These confined modes can be tuned to match the Raman excitation wavelength, optimizing the enhancement of scattered signals. Moreover, at the edges of the photonic bandgap of the photonic crystal, the group velocity of light is reduced, leading to the “slow light” effect, which can increase the interaction time between light and analyte molecules.^219^

WGM and waveguide resonators (Fig. 4d), such as semiconductor microspheres, microdisks, microrings, and microstripes, utilize total internal reflection to confine light along the curved surface of the structure.^220–225^ This confinement creates high-*Q* optical resonances, significantly amplifying the evanescent electromagnetic fields at the resonator's surface. The analyte molecules adsorbed onto these surfaces interact with the fields over the entire length of WGM, or waveguide, which can potentially lead to a higher Raman signal output. These structures are particularly appealing because they provide the building blocks for the emerging area of the photonic chip platform.^198,226–228^

The study of these diverse photonic structures highlights the versatility and potential of non-plasmonic materials for SERS applications. Further advancements in their design and integration into practical devices can potentially enhance their applicability across various sensing fields.

## 5. Semiconductor SERS nanostructures

After reviewing the fundamental principles of SERS substrates, the properties of semiconductor materials, and the various types of optical resonances, we now focus on the key semiconductor nanostructures used in SERS.

### 5.1. Randomly grown nanostructures

**Fig. 5:**
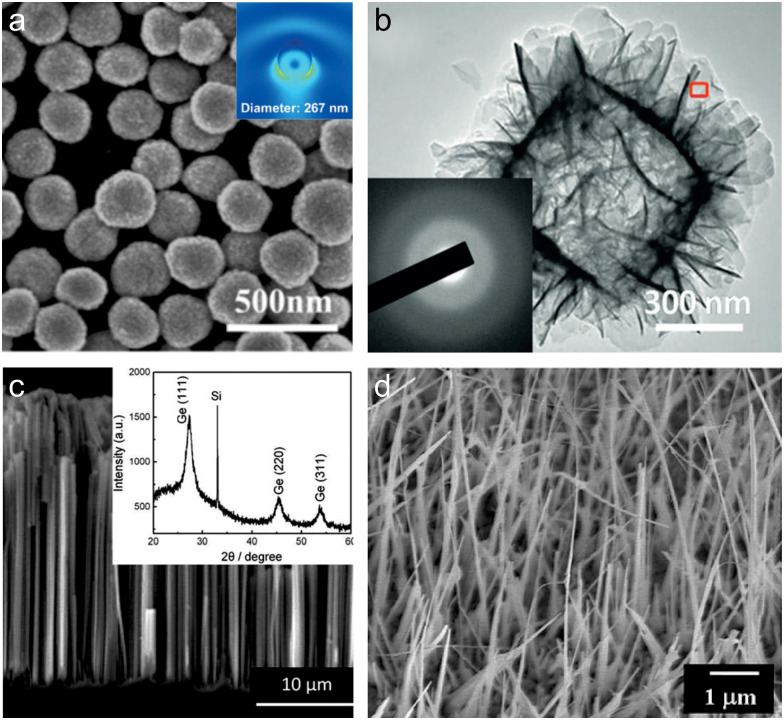
Examples of randomly grown semiconductor-based substrates for SERS applications: (a) SEM image of nanospheres with an inset showing the electric near-field distribution, demonstrating the “hot spots” due to Mie resonances. (b) SEM image of amorphous ZnO NPs. (c) Cross-sectional SEM image of Ge nanostructures, with the inset showing XRD patterns confirming their crystalline structure. (d) SEM images of the as-grown CuO nanowires. (a) (2019) Adapted with permission from ref. 50. Copyright 2019, John Wiley and Sons. (b) (2017) Adapted with permission from ref. 107. Copyright 2017, John Wiley and Sons. (c) (2011) Adapted with permission from ref. 130. Copyright 2011, American Chemical Society. (d) (2012) Adapted with permission from ref. 232. Copyright 2012, Elsevier.

First attempts to create SERS substrates based on semiconductor microarrays were based on randomly grown materials.^36,229^ They represent a versatile and cost-effective class of SERS substrates, leveraging the unique optical and electronic properties of semiconductors while avoiding complex lithographic fabrication. The most direct and simple way to create nanotextured SERS substrates is to use NPs (Fig. 5(a and b)) due to the ease of their fabrication and application.^31,36,50,92,95–97,104,107–111,115,116,192,230,231^ For example, the work by Hayashi *et al.* was one of the first experimental studies to show that semiconductor NPs can enhance Raman scattering.^36^ Using copper phthalocyanine (CuPc) molecules as probe analytes, they observed Raman signal enhancement with increasing GaP particle sizes. The maximum EF reached 700 for particles of 106 nm in size, which was explained by the EE mechanism due to the presence of Mie resonances. Early studies of NPs for SERS were mainly focused on wide-bandgap oxides such as TiO_2_ and ZnO, where Raman enhancement was primarily attributed to CT interactions between the semiconductor and adsorbed molecules.^96,103,104^ Since then, semiconductor NP SERS substrates have evolved significantly, with various materials demonstrating promising SERS activity. Further theoretical and experimental studies have shown that, in resonant semiconductor NPs, both mechanisms (CE and EE) make a combined contribution to the enhancement of the SERS signal.^51^ This was first observed in Cu_2_O NPs by C. Qiu *et al.* and L. Jiang *et al.*^108,109^ The former investigated porous Cu_2_O NPs and their aggregates and showed the possibility of obtaining an EF of ∼10^4^ and a sensitivity of up to 8 × 10^−6^ M. In the latter paper, the SERS activity of Cu_2_O NPs was investigated in more depth, revealing the mechanisms of both CE and EE mechanisms through density functional theory (DFT) calculations and finite difference time domain (FDTD) simulations. DFT calculations confirmed that the HOMO was localized on both the molecule and Cu_2_O, while the LUMO resided predominantly on Cu_2_O, confirming the presence of PICT. The CT contribution was estimated to provide EF ∼10^3^, making it the dominant mechanism. Additionally, FDTD simulations revealed localized electromagnetic field enhancement at the surface of Cu_2_O NPs and in NP dimers with small interparticle gaps, leading to an additional EF of ∼10^2^–10^3^. In the following years, the possibilities of using other materials were thoroughly studied in order to find ways to further improve the SERS enhancement of semiconductor NPs.^50,92,95,97,107,110,111,115,192,230,231^

Despite all the advantages of semiconductor NPs for SERS, their limitations, such as lower enhancement factors compared to metals, and difficulties in achieving uniform signal reproducibility, have driven researchers to explore alternative semiconductor nanostructures. Among these, nanowhiskers, nanorods and nanotubes have emerged as promising candidates due to their anisotropic geometry.^98,99,119,233–237^ Unlike spherical NPs, which have limited hot spot formation, they can generate intense electromagnetic fields at tips and along edges, improving the EE mechanism.^234,238–243^ In addition, a higher surface area to volume ratio increases the number of adsorbed molecules per unit substrate area, resulting in a stronger SERS signal. Initially, first studies were focused on isolated nanowires.^30,244^ However, because the overall efficiency of such structures was low, researchers switched to their arrays (Fig. 5(c and d)).^130,232^ For example, Wang *et al.* showed that vertically grown Si and Ge nanowire arrays with the diameter range of 100–250 nm can serve as efficient SERS substrates (Fig. 5a and b).^130^ The authors demonstrate that these structures can effectively enhance Raman signals through CT processes. By aligning the conduction and valence bands of the semiconductor substrates with the molecular energy levels of probe analytes such as rhodamine 6G, N719 dye, and 4-aminothiophenol, significant SERS sensitivities (10^−6^ M, 10^−5^ M, and 10^−3^ M, respectively) were achieved. In this work, it was shown that it is possible to improve the efficiency of CT processes between probe molecules and Si (Ge) by surface passivation of the samples, which showed that CE mechanisms can be utilized in single-atom semiconductors as SERS substrates.

However, the lack of precise control over size, shape, and spatial distribution leads to variability in SERS performance of the randomly grown nanostructures, making reproducibility a key challenge. While such structures offer high surface area, scalability, and ease of fabrication, their non-uniformity limits their suitability for applications requiring highly consistent signal enhancement.

### 5.2. Ordered nanostructures

To overcome the limitations of randomly grown nanostructures, researchers have increasingly turned to ordered semiconductor nanostructures, which offer greater control over size, shape, periodicity, and spatial distribution. Unlike their randomly formed counterparts, ordered structures enable the precise tuning of optical resonances, such as Mie modes and photonic bandgap effects, leading to more predictable and reproducible SERS enhancement.^63,199,245^ By carefully designing the arrangement of nanostructures, the electromagnetic field distribution can be optimized, creating well-defined electromagnetic hot spots that significantly improved Raman signal intensity.^26,49,51,52,58,231^

**Fig. 6:**
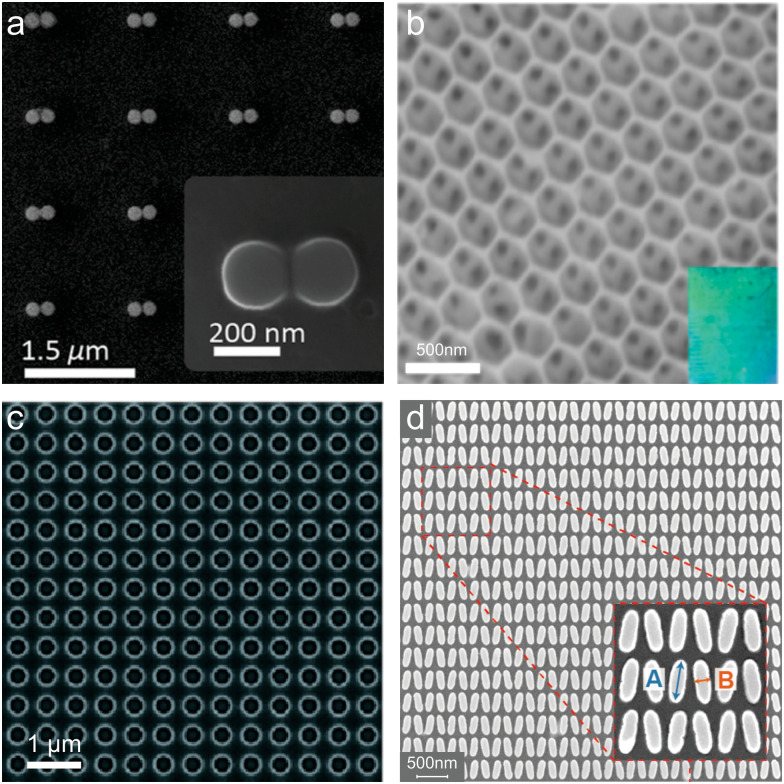
Ordered nanostructured platforms for enhanced SERS applications: (a) SEM image of the Si dimer nanoantennas. (b) SEM image of a plasmon-free TiO_2_ photonic microarray, showing its periodic structure designed for enhanced light–matter coupling. (c) SEM image of the Si_3_N_4_ BIC photonic crystal. (d) Metasurface pattern featuring elliptical nanostructures, designed to exploit BICs for enhanced light–matter interactions. (a) (2018) Adapted with permission from ref. 35. Copyright 2018, American Chemical Society. (b) (2014) Adapted with permission from ref. 91. Copyright 2014, American Chemical Society. (c) (2018) Adapted with permission from ref. 33. Copyright 2018, American Chemical Society. (d) (2024) Adapted with permission from ref. 34. Copyright 2024, John Wiley and Sons.

An important class of semiconductor SERS substrates are nanostructures based on Mie-resonant cylindrical and spherical nanoantennas.^63^ Such nanostructures may be of interest as, for example, substrates for microfluidic chips and biosensors.^5–8^ However, as mentioned earlier, single semiconductor nanoantennas amplify electromagnetic fields too weakly. Therefore, one of the solutions to this problem is to create structures with nanogaps between them. For example, the studies by Caldarola *et al.*^32^ and Cambiasso *et al.*^35^ (Fig. 6a) both focus on dielectric dimer nanoantennas as effective platforms for enhancing SERS. These works emphasize to use the advantages of high-refractive-index materials such Si, and demonstrate that dielectric nanoantennas can efficiently confine electromagnetic fields in nanoscale hot spots, leading to significant Raman signal amplification with an EF of approximately 10^3^, while maintaining ultra-low heat conversion. In turn, Wang *et al.* went further and showed that anapole modes may be the key to further improvement of semiconductor nanostructures for SERS.^246^ They investigated how the number of interacting nanoantennas having anapole modes affects the SERS enhancement. It was found that with an increase in the number of interacting nanoantennas from 3 to 7, the electromagnetic field enhancement in the nanogaps first increases (up to 60–70 times). However, already for an octomer, the electric field enhancement drops sharply. It has been shown that theoretically it is possible to obtain field enhancement factors of approximately 3000 to 5000. Despite the results achieved in this work, further experimental studies of semiconductor nanoantennas for SERS are required.

Similar to the case of randomly grown nanostructures, larger nanostructures are needed to further improve efficiency, signal-to-noise and surface-to-volume ratio, and scalability. Photonic crystal structures and metasurfaces can offer such properties, like high field enhancement and light confinement, resonance tunability, and precise fabrication of large-scale nanostructures by common techniques. For example, Qi *et al.* in 2014 have demonstrated the use of plasmon-free semiconductor TiO_2_ inverse opal photonic microarrays (Fig. 6b) as an innovative platform for SERS with the dual enhancement mechanism.^91^ They achieved highly sensitive SERS detection with an EF of ∼10^4^ and decreased the detection limit (up to 6 × 10^−6^ M) without the use of the hot spot effect by optimizing light–matter coupling through the structural design of the photonic microarray (Fig. 5d). The work highlighted the critical role of photonic band gap engineering, where the position of the band gap relative to the excitation wavelength was shown to govern the SERS sensitivity. Specifically, aligning the band gap edge with the laser wavelength resulted in a slow light effect, significantly enhancing light–matter interactions and Raman scattering intensity. In recent years, BIC metasurfaces have become a promising platform for highly sensitive SERS substrates due to their high and controllable *Q*-factors.^33,34,247^ BICs can confine electromagnetic energy in non-radiative localized states, significantly enhancing the interaction between incident light and analyte molecules. Unlike conventional semiconductor SERS substrates, BIC-based metasurfaces provide exceptional control over resonance conditions by engineering structural parameters and asymmetries, which can optimize Raman signal amplification. For example, Romano *et al.* showed near 10^3^-fold increase in fluorescence emission and Raman scattering intensity only due to the local electric field enhancement for a Si_3_N_4_-based BIC photonic crystal (Fig. 6c).^33^ The study by Chen *et al.* presents a BIC structure with strong-coupling effects, which opens up a photonic bandgap with a large Rabi splitting of ≈105 meV.^247^ It was found that theoretical SERS EF is angle dependent for this structure and can reach up to ∼10^17^, surpassing even metal-based SERS counterparts. Another significant advancement in this work is the integration of transformer-based deep learning for rapid metasurface optimization, replacing time-consuming electromagnetic simulations with AI-driven predictions of metasurface parameters for a given enhancement target. This AI-assisted approach enables real-time structure optimization, facilitating large-scale fabrication and implementation in practical sensing devices. In another work, Hu *et al.* demonstrated an approach to enhance SERS using both, EE and CE, mechanisms of TiO_2_-based BIC-driven metasurfaces (Fig. 6d).^34^ These semiconductor nanostructures achieved a high degree of electromagnetic field enhancement (|*E*/*E*_0_|^2^ ≈ 10^3^) and spectrally tunable absorption, addressing the conventional limitations of semiconductor SERS substrates such as weak EM enhancement and limited light–matter interactions. By incorporating quasi-BIC resonances through a symmetry-breaking designs, such as tilting ellipses or offsetting disk-hole structures, the study maximized light confinement and CT effects for improved Raman signal detection. TiO_2_ metasurfaces demonstrated a detection sensitivity for methylene blue down to 10^−8^ M, significantly surpassing previous benchmarks for TiO_2_-based SERS platforms.^91,92^ Moreover, the quasi-BIC resonances provided precise spectral tuning across visible wavelengths, allowing resonance alignment with commonly used laser sources (532 nm, 633 nm, and 785 nm) and enhancing PICT mechanisms. The integration of BIC-driven light manipulation and CE mechanisms is an example of a synergistic strategy for achieving high-performance SERS.

### 5.3. Applications

Semiconductor nanostructures have expanded the toolbox of SERS, enabling robust sensors. In biosensing, semiconductor nanostructures based on ZnO, TiO_2_, and MoS_2_ provide highly sensitive detection of biomarkers, proteins, and DNA through enhanced CT interactions and excitonic effects, enabling applications in disease diagnostics and biomedical research.^138,231,248–250^ Semiconductors have also been applied for chemical sensing and trace detection of environmental pollutants, toxins, and industrial contaminants with high specificity.^97,98,138,249,251–259^

As a future perspective, the integration of intelligent computational algorithms (artificial intelligence/machine learning) and microfluidic technologies with semiconductor SERS is a promising way to achieve faster and more accurate sensors, significantly expanding analytical capabilities in clinical diagnostics and environmental science.^247,260^ Moreover, an encouraging direction is the creation of tunable SERS substrates based on reconfigurable semiconductor metasurfaces and SERS field-effect transistors.^261–263^ Such sensors are not just passive devices, but can be actively optimized in real time.

## 6. Conclusions

We have reviewed recent advancements in the development of semiconductor metasurfaces for SERS, focusing on their ability to integrate optical and chemical enhancement mechanisms. Semiconductor substrates address the limitations of traditional plasmonic platforms through mechanisms such as Mie resonances and BIC, which enable localization and enhancement of strong electromagnetic fields. At the same time, semiconductor nanostructures can improve existing semiconductor SERS metasurfaces by engineering the semiconductor band gap, which facilitates efficient CT processes and enhances the chemical contribution to SERS. We analyzed the key design strategies for semiconductor substrates, emphasizing the importance of structural optimization and material selection. Controlling parameters such as the size, shape, periodicity, and symmetry of nanostructures can help to achieve strong field confinement and efficient resonance conditions. Material selection is equally critical, as the choice of semiconductors like TiO_2_, ZnO, or Si allows for tuning optical and electronic properties, including a refractive index and a bandgap, to enhance both electromagnetic and chemical contributions. These strategies enable the precise alignment of resonance wavelengths with excitation sources and analyte characteristics, providing robust and tailored SERS platforms for various sensing applications. Despite their significant potential, semiconductor materials face several challenges that limit their widespread adoption. For instance, the scalability of high-quality and reproducible semiconductor nanostructures remains a significant barrier, particularly for industrial-scale applications. Additionally, achieving an optimal balance between EE and CE mechanisms demands precise control over material properties and nanostructure design. Furthermore, some semiconductor materials exhibit limited enhancement factors compared to plasmonic counterparts, necessitating further optimization or the development of hybrid approaches. Long-term chemical and thermal stabilities in diverse environments, such as biomedical sensing and harsh industrial conditions, also require improvement. Finally, the integration of semiconductor SERS substrates with optical or electronic devices for seamless functionality is still in its early stages.

Looking ahead, several promising directions can address these limitations. Innovation in materials, such as 2D semiconductors, doped oxides, and hybrid systems, could significantly enhance both CE and EE contributions. Advances in fabrication techniques, including self-assembly, 3D printing, and machine learning-guided design, hold great potential for scalable and precise nanostructure production. Interdisciplinary integration of SERS with complementary techniques, such as fluorescence or mass spectrometry, could pave the way for multifunctional sensing platforms. Tailoring semiconductor SERS substrates for specific applications, such as wearable sensors, real-time environmental monitoring, and *in vivo* diagnostics, could further enhance their impact. Moreover, focusing on eco-friendly and cost-effective materials and fabrication methods will ensure sustainability and accessibility. These developments highlight a bright future for semiconductor-based SERS, establishing them as a cornerstone for next-generation sensing technologies.

## Conflicts of interest

There are no conflicts to declare.

## Data Availability

No primary research results, software or code have been included and no new data were generated or analysed as part of this review.
